# Effect of Experimentally Induced Hepatic and Renal Failure on the Pharmacokinetics of Topiramate in Rats

**DOI:** 10.1155/2014/570910

**Published:** 2014-06-09

**Authors:** Kamal M. Matar, Yasin I. Tayem

**Affiliations:** ^1^Department of Pharmacology and Therapeutics, Faculty of Pharmacy, Kuwait University, P.O. Box 24923, Safat 13110, Kuwait; ^2^Department of Physiology and Pharmacology, School of Medicine, Al-Quds University, Abu Dees, West Bank, Jerusalem, Palestine

## Abstract

We aimed to investigate the effect of induced hepatic and renal failure on the pharmacokinetics of topiramate (TPM) in rats. Twenty-four Sprague-Dawley rats were used in this study. Renal or hepatic failure was induced by a single i.p. dose of 7.5 mg/kg cisplatin (*n* = 8) or 0.5 mL/kg carbon tetrachloride (CCl_4_) (*n* = 8), respectively. Three days after cisplatin dose or 24 h after CCl_4_ dose, the rats were administered a single oral dose of 20 mg/kg TPM. The plasma samples were quantified by LC-MS/MS method. Compared to control, plasma concentration-time profile in CCl_4_-treated and, to a lesser extent, in cisplatin-treated rats decreased more slowly particularly in the elimination phase. TPM oral clearance (CL/F) in CCl_4_-treated group was significantly lower than that in control (*P* < 0.001), whereas AUC_0−*∞*_, T1/2, and Vd/F were significantly higher in CCl_4_-treated rats compared to the control (*P* < 0.01). The CL/F was not significantly different between cisplatin-treated rats and control (*P* > 0.05). However, in cisplatin-treated rats, the T1/2 and Vd/F were significantly higher than that in the control group (*P* < 0.01). Both conditions failed to cause a significant effect on *C*_max_ or *T*_max_. The present findings suggest that induced hepatic or renal failure could modify the pharmacokinetic profile of TPM in the rat.

## 1. Introduction

Topiramate (TPM) is a structurally novel broad spectrum anticonvulsant that is effective in the treatment of several types of epilepsy including partial onset [1] and primary generalized seizures [2]. TPM, which was originally designed as an oral hypoglycemic agent [3], was found to be an effective and tolerable antiepileptic drug, both as monotherapy and as add-on, in children and adults [4].

The pharmacokinetic profile of TPM in humans has been extensively characterized. Following oral administration, TPM is rapidly absorbed with high bioavailability (~80%) and low binding to plasma proteins [5]. Peak plasma concentration is reached within 1–4 h after administration [5]. In the dose range of 100 to 1200 mg, the mean apparent volume of distribution (Vd) is between 0.6 and 1.0 L/kg [5]. At steady-state concentration, renal clearance of this drug is 1.02 L/h [6] and its elimination half-life (T1/2) varies from 20 to 30 h [5]. In all species, TPM is predominantly excreted unchanged in urine [6].

Liver metabolic pathways of TPM in humans and animals are similar and involve hydroxylation or hydrolysis of isopropylidene groups [7]. Out of these hepatic metabolizing systems, cytochrome P450 (CYP) enzymes play a major role in the biotransformation and inactivation of this agent [5]. In general, alterations in the activity of CYP, as a result of liver diseases, decrease drug metabolism thus requiring dosage adjustment and therapeutic drug monitoring (TDM) [8]. The mechanisms by which liver diseases modify drug pharmacokinetics occur alone or in combination and encompass, in addition to altering CYP activity, changes in hepatic blood flow and binding of drugs to plasma proteins [8]. The latter influences the processes of drug distribution and elimination. The ultimate effect of these factors leads to a decrease in plasma clearance and an increase in the extent of absorption of drugs by decreasing their first pass effect following oral administration. In addition, patients with advanced liver disease often suffer from deterioration in renal function and dose adjustment becomes necessary even for drugs which are predominantly excreted unchanged in urine [9].

Since kidney is the major route of drug excretion from the body, any deterioration in renal function, even if it is mild, dramatically influences the pharmacokinetic characteristics of a drug and its metabolites. In fact, this is one of the most important factors which influence patient's response to an administered drug. Various pharmacokinetic parameters can be altered in response to renal impairment including absorption [10], bioavailability [11], Vd [12], and metabolism [13]. These alterations often require dose adjustment to avoid drug accumulation and serious toxicity. For these reasons, pharmacokinetic studies in renal impairment patients are an integral part of new drug development programs [14]. A key objective of these studies is to determine if disruption in renal function can alter pharmacokinetics of a drug sufficiently to require dosage adjustment.

To our knowledge, the effect of renal and hepatic failure on the pharmacokinetics of TPM has not been fully elucidated yet. For this reason, the present investigation was carried out to provide insight into the influence of compromised liver and kidney function on the pharmacokinetic profile of TPM in rats.

## 2. Materials and Methods

### 2.1. Chemicals and Reagents

The commercial formulation of TPM, Topamax (Janssen-Cilag, Schaffhausen, Switzerland) containing 100 mg TPM, was locally purchased from a drug store. CCl_4_ and carboxymethylcellulose (CMC) were purchased from Sigma-Aldrich Company (Poole, UK). Cisplatin, the commercial formulation of cisplatin (Ebewe Pharma, GmbH, Unterach, Austria) containing 50 mg/100 mL cisplatin injection, was locally purchased. TPM-d_12_ (internal standard “IS” for TPM analysis) was purchased from BDG Synthesis (Wellington, New Zealand). Water was purified using a Milli-Q water device (Millipore, Bedford, MA, USA). All other chemicals and reagents were of analytical grade and solvents were of HPLC grade. TPM was freshly prepared from tablet formulation suspended in 0.5% CMC. CCl_4_ was dissolved in olive oil (1 : 1 v/v) and cisplatin was used directly from a bottle for injection (50 mg/100 mL) without further dilution. Serum creatinine (Scr) and aspartate aminotransferase (AST) were measured spectrophotometrically using diagnostic kits purchased from Human GmbH (Wiesbaden, Germany).

### 2.2. Ethical Approval

Ethical approval for conducting this study was provided by the local Experimental Animal Resource Center Ethics Committee, Health Sciences Center, Kuwait University, and it was in compliance with the Helsinki Declaration for Ethical Principles of Medical Research.

### 2.3. Animals and Treatment

Male Sprague-Dawley rats (*n* = 24), weighing between 180 and 230 g, were used in this study and were housed in a pathogen-free facility. The rats were acclimatized at least for one week before experiments in an animal facility in which the appropriate temperature, humidity, and light cycle (6:00 A.M.–6:00 P.M) were maintained, with* ad libitum* access to food and water. The rats were divided into 3 groups (*n* = 8 each). Group 1 was administered a single oral dose of 20 mg/kg TPM. Group 2 was injected a single i.p. dose of 7.5 mg/kg CP followed, seventy-two hours later, by a single oral dose of 20 mg/kg TPM. Group 3 was injected a single i.p. dose of 0.5 mL/kg CCl_4_ followed, twenty-four hours later, by a single oral dose of 20 mg/kg TPM. Biochemical parameters including Scr and AST for the three groups were measured using appropriate diagnostic kits before the pharmacokinetic study was conducted.

For the three groups, blood samples (~0.3 mL each) were collected in preheparinized Eppendorf tubes from light ether-anesthetized rats just before and at 0.25, 0.5, 1.0, 1.5, 2, 4, 6, 8, 12, 18, and 24 h following TPM administration. The blood samples were collected following the previously reported procedure [15]. The blood samples were immediately centrifuged at 9000 ×g for 10 min. The plasma samples were then separated and aliquots of 100 *μ*L were kept frozen at −80°C pending analysis.

### 2.4. Plasma Samples Analysis

TPM plasma samples were analyzed using a previously described tandem mass spectrometric (LC-MS/MS) method [16]. The linear range of the method was 0.5–30 *μ*g/mL and the lower limit of quantification was 0.5 *μ*g/mL. The intra- and interrun precisions, as measured by relative standard deviations (RSD %), of the method were less than 8%.

Prior to the assay, the frozen rat plasma samples, calibrators, and quality control samples were gently thawed at ambient temperature and then vortex-mixed for 30 s before extraction. To each 100 *μ*L of plasma sample, 25 *μ*L of IS (100 *μ*g/mL) was added and vortex-mixed for 30 s. To each tube, 25 *μ*L of ammonium acetate (10 mM) and 1 mL of ether were added and the mixture was then centrifuged at 9000 ×g for 10 min. The organic layer was separated, evaporated to dryness, and then reconstituted with 100 *μ*L of the mobile phase; acetonitrile: 0.1% triethylamine (80 : 20, v/v). A 10 *μ*L of this solution was then injected into the LC-MS/MS system.

### 2.5. Pharmacokinetics and Statistical Analyses

TPM pharmacokinetic parameters were estimated by standard noncompartmental methods using Kinetica software, version 5.1 (Thermo Fisher Scientific, USA). The maximum plasma concentration (*C*_max⁡_) and time needed to attain this concentration (*T*_max⁡_) were directly obtained from the drug plasma profiles; the drug plasma elimination half-life (T1/2) values were calculated as Ln2/kel, where kel is the elimination rate constant. The area under the plasma concentration-time curves (AUC_0-*t*_) was calculated from the measured data points from time zero to time of last quantifiable concentration by the linear trapezoidal rule and the area under the plasma concentration-time curves extrapolated to infinity (AUC_0-*∞*_) was calculated using the equation: AUC_0-*∞*_ = AUC_0-*t*_ + C*/kel, where C* is the last quantifiable drug plasma concentration. Oral body clearance (CL/F) was calculated as CL/F = Dose/AUC_0-*∞*_ and the volume of distribution (Vd/F) was calculated as Vd/F = (CL/F)/kel. The pharmacokinetic parameters were presented as mean ± SD. Differences between the pharmacokinetic parameters of TPM among the groups were considered statistically significant if *P* < 0.05 using Mann-Whitney* U* test (nonparametric for two independent samples). The statistical analysis was performed using the statistical package for social sciences (SPSS) software, version 20 (SPSS Inc., Chicago, IL, USA).

## 3. Results

**Figure 1:**
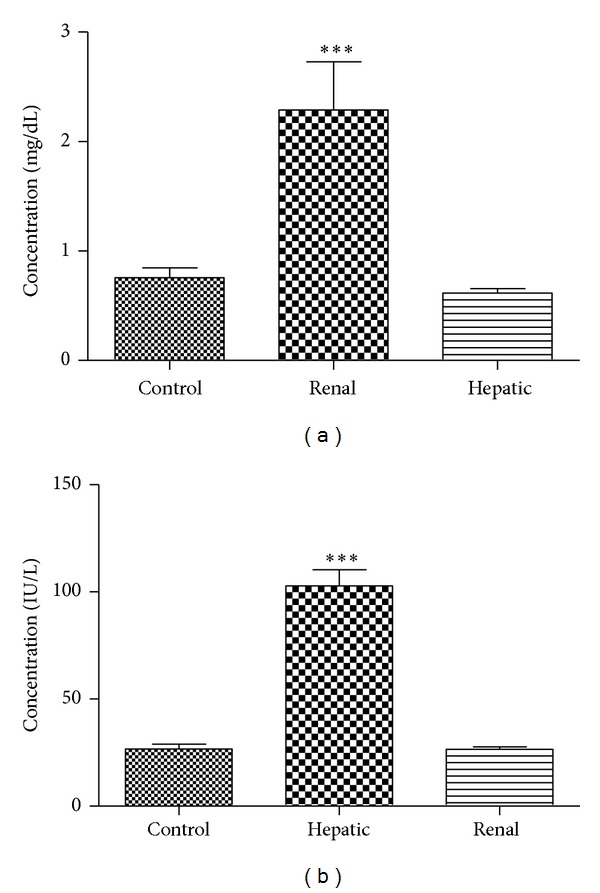
Serum creatinine (a) and aspartate transaminase (AST) (b) in control and experimentally induced hepatic or renal failure rats. Data presented are mean ± SE in 16 rats. ***P < 0.0001, significantly different from control group.

Biochemical parameters in cisplatin-treated or CCl_4_-treated rats are depicted in Figure 1. Cisplatin pretreatment significantly increased serum creatinine levels (Figure 1(a)) in contrast to control rats (*P* < 0.0001). There was no difference in creatinine levels between CCl_4_-treated and control rats (*P* > 0.05). On the other hand, CCl_4_-treated rats significantly increased AST levels in comparison to control rats (*P* < 0.0001), and there was no significant difference in AST levels between control and cisplatin-treated rats (*P* > 0.05), as shown in Figure 1(b).

**Figure 2:**
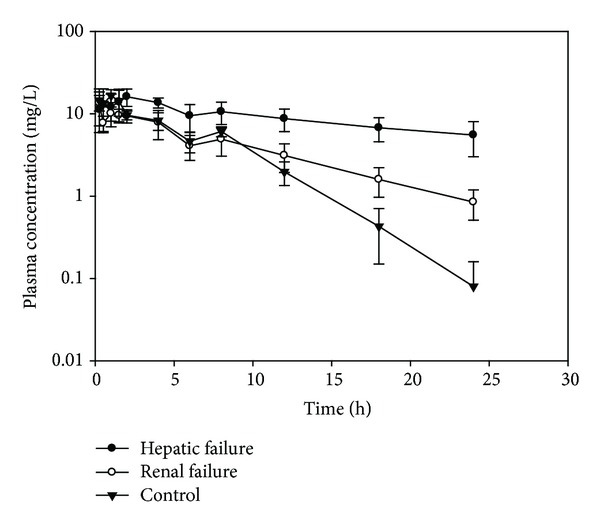
Plasma concentration-time profile of TPM following an oral dose of 20 mg/kg to control and experimentally induced hepatic or renal failure rats (*n* = 8).

Mean plasma concentration-time profile for TPM following an oral dose of 20 mg/kg to CCl_4_-treated, cisplatin-treated, or control rats is demonstrated in Figure 2 and the mean pharmacokinetic parameters of TPM after an oral administration of 20 mg/kg TPM dose to the same groups are presented in Table 1. As shown in Figure 2, the plasma concentration-time profiles following an oral TPM dose (20 mg/kg) were different between CCl_4_-treated, cisplatin-treated, and control rats. The profile in CCl_4_-treated and cisplatin-treated rats was decreased more slowly than that in control rats, being significantly higher in the elimination phase compared to that in control rats. However, the impact was more pronounced in CCl_4_-treated rats than in cisplatin-treated rats. TPM oral clearance (CL/F) in CCl_4_-treated rats was significantly lower than that in control rats (*P* < 0.001) and was decreased by 71% in comparison to the control group, as shown in Table 1. This results in significantly higher values of AUC_0-*∞*_, T1/2, and Vd in this group in comparison to the control group (*P* < 0.01). On the other hand, CL/F was not significantly different between cisplatin-treated rats and control group (*P* > 0.05), as shown in Table 1. However, the T1/2 and Vd in cisplatin-treated rats were significantly higher than in control group (*P* < 0.01).

## 4. Discussion

Patients with epilepsy may suffer from concurrent renal or hepatic diseases that can modify the pharmacokinetic profiles of antiepileptic drugs (AEDs). The present study systematically investigated the influence of compromised renal or hepatic function on TPM pharmacokinetics employing rat models of kidney or liver failure using a validated tandem mass spectrometric method (LC-MS/MS) [16]. To the best of our knowledge, this is the first study which examined the impact of these two conditions on the way body handles TPM dose. Our data demonstrated that both experimentally induced renal and hepatic failure modified the pharmacokinetic profile of this AED leading to prolongation in its T1/2. If the findings of the present study are extrapolated to humans, this may emphasize that dose adjustment of TPM may be required when this drug is administered to epileptic patients with renal or hepatic failure. That is, therapeutic levels should be achieved in these cases by administering smaller maintenance doses or by increasing dosing intervals to avoid accumulation of this agent in the body.

Our data demonstrated that TPM *C*_max⁡_ and *T*_max⁡_ values were not significantly altered by experimentally induced hepatic or renal failure. This perhaps indicates that TPM's absorption was not significantly changed in both cases. However, there was a significant increase in the drug's AUC_0-*∞*_ in CCl_4_-treated rats compared to cisplatin-treated or control rats. This could be due to reduced oral clearance in this group in comparison with the other two groups. In addition, the observed significant increase in AUC_0-*∞*_ in CCl_4_-treated rats could be related to portal-systemic shunting, which occurs frequently in advanced liver disease. This complication may substantially decrease drug metabolism and result in a significant decrease in nonrenal clearance [17]. In accordance with our findings, Brockmöller and coworkers investigated possible changes in the pharmacokinetics of levetiracetam in patients with liver cirrhosis. Similar to TPM, this newer AED is mainly excreted unchanged in urine and is not metabolized by the liver. The investigators found that, in patients with severe cirrhosis, the drug clearance was reduced by 43% and plasma T1/2 was significantly prolonged compared with healthy subjects. The authors measured renal clearance of this AED and found that it was dramatically reduced. In addition, they reported that the decrease in renal clearance was correlated with the deterioration in renal function [9].

Based on our current findings, it is interesting to note that the mean plasma concentration-time profile of TPM, particularly in the elimination phase, decreased slowly. This observation was seen in both renal and hepatic impairment models, but it was more profound in the latter. This was accompanied by an increase in the elimination T1/2 of the drug and a significant decrease in its CL/F. It is more likely that the slower fall in TPM levels in CCl_4_-treated rats is related to a decrease in nonrenal clearance rather than an increase in the drug's bioavailability. TPM is predominantly excreted unchanged in urine [6], so theoretically the drug plasma concentration was expected to be higher in the renal failure group. However, the decrease in the oral clearance of the drug in CCl_4_-treated group could be attributed to the development of renal dysfunction in the rats which suffered from hepatic failure [18]. Paradoxically, however, this decline in kidney function did not cause rise in serum creatinine since hepatic impairment is normally accompanied by reduced serum creatinine level and resultant overestimation of creatinine clearance. Therefore, measurement of renal clearance rather than total clearance in this setting would be more useful. Compared to those who have normal renal function, TPM clearance in patients with moderate and severe renal impairment is reduced by 42 and 54%, respectively [19]. Despite the fact that TPM is not extensively metabolized, it was previously demonstrated that moderate-to-severe liver impairment can decrease its oral clearance by 26% [6]. In our study involving experimentally induced renal failure rats, we observed that TPM elimination T1/2 was modestly increased, whereas the Vd/F was significantly elevated (Table 1). The significant increase in Vd/F in this group is most likely a resultant of decreased bioavailability. However, this postulation needs further investigation. Given that TPM is normally renally excreted, the explanation for the absence of a considerable increase in the oral clearance of the drug in cisplatin-induced renal failure rats is presumably related to the significant elevation of Vd/F which indicated that most of the drug was rather redistributed in body fluids and was not available for elimination. In accordance with our findings, Glue and coworkers evaluated the effect of renal impairment on single-dose pharmacokinetics of the newer AED, felbamate. The investigators showed that, compared to controls, total clearance of felbamate decreased while T1/2 and AUC_0-*∞*_ values increased in subjects with renal dysfunction. The magnitude of these changes was correlated with the degree of renal dysfunction. In contrast to our findings, however, Vd/F was lower in renal dysfunction subjects [20]. In another study, Lal and coworkers described a population-based pharmacokinetic study of gabapentin enacarbil in patients with varying degrees of renal dysfunction. The authors reported that gabapentin CL/F was proportionally related to creatinine clearance [21]. Similar to hepatic dysfunction, our data on experimentally induced renal failure indicated that dosage adjustment may be necessary in patients with compromised renal function if the present findings are extrapolated to human beings.

Based on the preceding discussion, some general observations can be made about the impact of experimentally induced liver and kidney failure on the way our body handles TPM. Overall, the findings of this study may suggest that TPM administration to patients suffering from these two conditions may result in prolongation in its elimination T1/2. These results emphasize the need for therapeutic drug monitoring of TPM in these settings to avoid drug accumulation and toxicity if the present findings are to be extrapolated to humans.

In conclusion, the findings of the present study suggest that the pharmacokinetic profile of TPM may be altered in the presence of compromised liver or kidney function. Further studies involving intravenous administration of TPM and including urine data are warranted to investigate the mechanisms underlying the slow fall in TPM levels in these two conditions.

## Figures and Tables

**Table 1:** Pharmacokinetic parameters of TPM in experimentally induced hepatic or renal failure rats.

Parameter	Control	Hepatic	Renal
*T*_max⁡_ (h)	0.71 ± 0.53	1.13 ± 0.82	0.38 ± 0.31
*C*_max⁡_ (*μ*g/mL)	17.98 ± 2.56	20.00 ± 4.77	14.43 ± 5.77
T1/2 (h)	2.57 ± 0.64	21.16 ± 14.61^a^	6.21 ± 0.43^b^
AUC_0−*∞*_ (*μ*g·h/mL)	93.44 ± 17.68	430.84 ± 258.77^a^	102.19 ± 33.35
Vd/F (L)	0.79 ± 0.10	1.36 ± 0.36^b^	1.92 ± 0.63^b^
CL/F (mL/h/kg)	220.78 ± 43.21	63.69 ± 39.28^a^	215.38 ± 75.69

Data are shown as mean ± SD for 8 rats.

^a^Significantly different (*P* < 0.001) from control.

^b^Significantly different (*P* < 0.01) from control.

## Abbreviations

AST: Aspartate aminotransaminase
CCl_4_: Carbon tetrachloride
TPM: Topiramate
T1/2: Elimination half-life
*C*_max⁡_: Maximum plasma concentration
*T*_max⁡_: Time needed to reach maximum plasma concentration
AUC_0-*∞*_: Area under the plasma concentration-time curve from time zero to infinity
Vd: Apparent volume of distribution
CL/F: Oral clearance
*F*: Bioavailability factor.

## References

[1] Kaminska A (2001). New antiepileptic drugs in childhood epilepsies: indications and limits. *Epileptic Disorders*.

[2] Gursahani R, Gupta N (2012). The adolescent or adult with generalized tonic-clonic seizures. *Annals of Indian Academy of Neurology*.

[3] Liang Y, Chen X, Osborne M, DeCarlo SO, Jetton TL, Demarest K (2005). Topiramate ameliorates hyperglycaemia and improves glucose-stimulated insulin release in ZDF rats and db/db mice. *Diabetes, Obesity and Metabolism*.

[4] Kholin AA, Zavadenko NN, Il’Ina ES (2013). Efficacy and safety of topiramate depending on patient’s age and forms of epilepsy. *Zhurnal Nevrologii i Psihiatrii imeni S.S. Korsakova*.

[5] Patsalos PN (2005). Properties of antiepileptic drugs in the treatment of idiopathic generalized epilepsies. *Epilepsia*.

[6] Sachdeo RC (1998). Topiramate: clinical profile in epilepsy. *Clinical Pharmacokinetics*.

[7] Caldwell GW, Wu WN, Masucci JA (2005). Metabolism and excretion of the antiepileptic/antimigraine drug, topiramate in animals and humans. *European Journal of Drug Metabolism and Pharmacokinetics*.

[8] Rodighiero V (1999). Effects of liver disease on pharmacokinetics. An update. *Clinical Pharmacokinetics*.

[9] Brockmöller J, Thomsen T, Wittstock M, Coupez R, Lochs H, Roots I (2005). Pharmacokinetics of levetiracetam in patients with moderate to severe liver cirrhosis (Child-Pugh classes A, B, and C): characterization by dynamic liver function tests. *Clinical Pharmacology and Therapeutics*.

[10] Craig RM, Murphy P, Gibson TP (1983). Kinetic analysis of D-xylose absorption in normal subjects and in patients with chronic renal failure. *Journal of Laboratory and Clinical Medicine*.

[11] Ohnhaus EE, Vozeh S, Nuesch E (1979). Absolute bioavailability of digoxin in chronic renal failure. *Clinical Nephrology*.

[12] St. Peter WL (2010). Improving medication safety in chronic kidney disease patients on dialysis through medication reconciliation. *Advances in Chronic Kidney Disease*.

[13] Wynne H (2005). Drug metabolism and ageing. *Journal of the British Menopause Society*.

[14] Flaharty KK, Grimm AM, Vlasses PH (1989). Epoetin: human recombinant erythropoietin. *Clinical Pharmacy*.

[15] Matar KM (2005). Influence of famotidine on verapamil pharmacokinetics in rats. *European Journal of Drug Metabolism and Pharmacokinetics*.

[16] Matar KM (2010). Therapeutic drug monitoring of topiramate by liquid chromatography-tandem mass spectrometry. *Clinica Chimica Acta*.

[17] Bosilkovska M, Walder B, Besson M, Daali Y, Desmeules J (2012). Analgesics in patients with hepatic impairment: pharmacology and clinical implications. *Drugs*.

[18] Li X, Hassoun HT, Santora R, Rabb H (2009). Organ crosstalk: the role of the kidney. *Current Opinion in Critical Care*.

[19] Stefan H, Feuerstein TJ (2007). Novel anticonvulsant drugs. *Pharmacology and Therapeutics*.

[20] Glue P, Sulowicz W, Colucci R (1997). Single-dose pharmacokinetics of felbamate in patients with renal dysfunction. *British Journal of Clinical Pharmacology*.

[21] Lal R, Sukbuntherng J, Luo W (2012). Clinical pharmacokinetics of gabapentin after administration of gabapentin enacarbil extended-release tablets in patients with varying degrees of renal function using data from an open-label, single-dose pharmacokinetic study. *Clinical Therapeutics*.

